# Revised 16S rRNA V4 hypervariable region targeting primers enhance detection of Patescibacteria and other lineages across diverse environments

**DOI:** 10.1093/ismeco/ycag141

**Published:** 2026-05-21

**Authors:** Huifeng Hu, Clemens Karwautz, Kalina Duszka, Thomas Karner, Isabella C Wagner, Christoph Grander, Wilhelm Grander, Laura Steinwidder, Lucilla Boito, Viktor Van de Velde, Marijn Bauters, Pascal Boeckx, David Seki, Bettina Glasl, Stefan Thiele, Hannes Schmidt, Joana Séneca, Michael Wagner, Petra Pjevac

**Affiliations:** Centre for Microbiology and Environmental Systems Science, University of Vienna, Djerassiplatz 1, Vienna 1030, Austria; Doctoral School in Microbiology and Environmental Science, University of Vienna, Universitätsring 1, Vienna 1010, Austria; Department of Functional and Evolutionary Ecology, Faculty of Life Sciences, University of Vienna, Djerassiplatz 1, Vienna 1030, Austria; Department of Nutritional Sciences, Faculty of Lifesciences, University of Vienna, Josef-Holaubek-Platz 2, Vienna 1090, Austria; Centre for Animal Nutrition and Welfare, University of Veterinary Medicine, Veterinärplatz 1, Vienna 1210, Austria; Faculty of Psychology, University of Vienna, Liebiggasse 5, Vienna 1010, Austria; Centre for Microbiology and Environmental Systems Science, University of Vienna, Djerassiplatz 1, Vienna 1030, Austria; Faculty of Psychology, University of Vienna, Liebiggasse 5, Vienna 1010, Austria; Department of Internal Medicine I, Gastroenterology, Hepatology, Endocrinology, and Metabolism, Medical University of Innsbruck, Innrain 52, Innsbruck 6020, Austria; Department of Internal Medicine, Hall State Hospital, Milserstrasse 10, Hall in Tirol 6060, Austria; Biobased Sustainability Engineering (SUSTAIN), Department of Bioscience Engineering, University of Antwerp, Antwerp 2020, Belgium; Biobased Sustainability Engineering (SUSTAIN), Department of Bioscience Engineering, University of Antwerp, Antwerp 2020, Belgium; Department of Environment, Faculty of Bioscience Engineering, Ghent University, Coupure Links 653, Gent 9000, Belgium; Department of Green Chemistry and Technology, Faculty of Bioscience Engineering, Ghent University, Coupure Links 653, Gent 9000, Belgium; Department of Environment, Faculty of Bioscience Engineering, Ghent University, Coupure Links 653, Gent 9000, Belgium; Department of Green Chemistry and Technology, Faculty of Bioscience Engineering, Ghent University, Coupure Links 653, Gent 9000, Belgium; Centre for Microbiology and Environmental Systems Science, University of Vienna, Djerassiplatz 1, Vienna 1030, Austria; Centre for Microbiology and Environmental Systems Science, University of Vienna, Djerassiplatz 1, Vienna 1030, Austria; Centre for Microbiology and Environmental Systems Science, University of Vienna, Djerassiplatz 1, Vienna 1030, Austria; Centre for Microbiology and Environmental Systems Science, University of Vienna, Djerassiplatz 1, Vienna 1030, Austria; Centre for Microbiology and Environmental Systems Science, University of Vienna, Djerassiplatz 1, Vienna 1030, Austria; Department of Laboratory Medicine, Medical University of Vienna, Währinger Gürtel 18-20, Vienna 1090, Austria; Joint Microbiome Facility, Medical University of Vienna and the University of Vienna, Djerassiplatz 1, Vienna 1030, Austria; Centre for Microbiology and Environmental Systems Science, University of Vienna, Djerassiplatz 1, Vienna 1030, Austria; Joint Microbiome Facility, Medical University of Vienna and the University of Vienna, Djerassiplatz 1, Vienna 1030, Austria; Center for Microbial Communities, Department of Chemistry and Bioscience, Aalborg University, Fredrik Bajers Vej 7H, Aalborg 9220, Denmark; Centre for Microbiology and Environmental Systems Science, University of Vienna, Djerassiplatz 1, Vienna 1030, Austria; Joint Microbiome Facility, Medical University of Vienna and the University of Vienna, Djerassiplatz 1, Vienna 1030, Austria; Environment and Climate Hub (ECH), University of Vienna, Augasse 2-6, Vienna 1090, Austria

**Keywords:** 16S rRNA gene amplicon sequencing, primer modification, Patescibacteria

## Abstract

Primer bias in 16S rRNA gene amplicon sequencing can distort microbial diversity estimates by underrepresenting key taxa. We introduce a modified primer pair (V4-EXT) targeting the hypervariable V4 region of bacterial and archaeal 16S rRNA genes, with improved *in silico* taxonomic inclusivity. To benchmark performance, we analyzed 938 samples from terrestrial, aquatic, and host-associated habitats, comparing microbial community profiles derived with V4-EXT and the currently most widely used V4-targeted primers. V4-EXT substantially improved the detection of Patescibacteria and other underrepresented lineages, such as Chloroflexi and Iainarchaeota, while enhancing recovery of novel amplicon sequence variants across sample types. Overall, V4-EXT provides broader taxonomic coverage and more inclusive microbial community profiles, particularly in high-diversity ecosystems such as groundwater and soils. We propose V4-EXT as a robust successor for comprehensive microbial community analysis across diverse habitats.

## Introduction

Gene-targeted amplicon sequencing remains the most widely used sequencing-based technique in microbial ecology and microbiome research. It enables low-cost, high-throughput analyses of microbial community composition and potential metabolic functions in diverse environments by targeting, amplifying, and sequencing phylogenetic or functional marker genes [1–6]. Despite its utility, amplicon sequencing has several well-recognized limitations, including effects of variability in gene copy number, primer bias, PCR stochasticity, reliance on reference databases for amplicon classification, and the compositional nature of the generated relative abundance data [7–9]. Among these, primer bias is an important and experimentally tractable source of systemic distortion. Mismatches between primers and target sequences can result in selective amplification and the consistent underrepresentation of entire microbial lineages. As a consequence, taxa may appear rare or absent in datasets despite being present in the community. Such biased datasets potentially skew diversity estimates, community composition profiles, and ecological interpretations [10, 11]. In this study, we focus specifically on primer inclusivity, with the aim of improving the detection and representation of taxa that so far have been systematically underrepresented.

Most studies analyzing microbial communities use broad-range primers that target the 16S rRNA phylogenetic marker gene. Since the widespread implementation of short-read sequencing, primers targeting conserved regions flanking short, hypervariable stretches (mainly V1-V2, V3-V4, V4, and V9), generating 200–350 bp amplicons, have become commonplace. The most commonly used among these are the bacterial and archaeal 16S rRNA V4 targeted primers 515F [10] and 806R [11], used by the Earth Microbiome Project and hereafter referred to as “V4-EMP” [1]. In a recent study, we demonstrated that a modification of these V4 primers significantly improved the detection of Patescibacteria, highlighting primer inclusivity as a critical determinant of observed community composition. By applying the optimized primer (hereafter referred to as “V4-CPR”) to a global collection of wastewater treatment plant (WWTP) samples, we revealed that Patescibacteria constitute a substantial and previously largely overlooked fraction of WWTP microbial communities [12].

Patescibacteria are a diverse and widespread group of bacteria that have been discovered only in the past decades and remain hard to isolate and cultivate. Patescibacteria inhabit a broad range of ecosystems, including groundwater, freshwater, soils, sediments, subsurface aquifers, wastewater treatment systems, and host-associated microbiomes. They generally exhibit an epibiontic lifestyle, parasitizing a wide diversity of microbial hosts. Metagenomics studies have revealed that they are especially highly abundant in groundwater ecosystems, where they can constitute 20%–50% of the total bacterial community [13], but they also constitute substantial fractions in WWTP [12], the human oral cavity [14], and diverse freshwater habitats [15]. Due to their highly divergent 16S rRNA genes [12], Patescibacteria are poorly covered by most primers commonly used for microbial community profiling, which prompted us to design the Patescibacteria-inclusive V4-CPR primer in our previous study. However, V4-CPR has only been experimentally evaluated with WWTP samples and has shown limited *in silico* coverage of some archaeal lineages that are typically low in abundance in WWTPs.

In the present study, we further increased the taxonomic inclusivity of the V4-targeted primers by improving coverage of archaea via reintroducing a single-base degeneracy in the forward primer (at position 523, A to M), while maintaining improved Patescibacteria detection. We evaluated the performance of this revised 16S rRNA V4 primer set, hereafter referred to as “V4 extended” or “V4-EXT,” by applying it to 938 samples from various sources, including soil, freshwater, and marine environments, as well as human and animal stool. Our sample source selection included habitats known for both high and low paterscibacterial or archaeal relative abundances, allowing for a comprehensive evaluation of *in situ* primer performance. We analyzed differences in community alpha diversity, community taxonomic profiles, and detected sequence novelty between datasets generated with the V4-EXT and V4-EMP primers. Our results demonstrate that the V4-EXT primers offer an expanded taxonomic detection range and improved sensitivity for Patescibacteria and other taxa previously underdetected due to primer bias. The use of V4-EXT results in more inclusive microbial diversity profiles, especially when applied to soil, freshwater, and ocean samples.

Based on these findings, we recommend using the V4-EXT instead of V4-EMP in future studies, as it enables more comprehensive microbial community profiling across different habitats.

## Materials and methods

### Sample collections and amplicon sequencing

We analyzed 938 samples from human and animal stool, groundwater, surface water, ocean, sponge, and soil collected in the framework of 12 independent research projects (Table S1). DNA extraction for soil samples from projects JMF-2401-02, JMF-2401-04, and JMF-2401-05 was performed using ~250 mg soil (wet weight) per sample and the Qiagen DNeasy PowerSoil Pro Kit with the addition of 15% of a 20% skimmed milk solution to the CD1 buffer. Samples from project JMF-2210-08 and JMF-2212-20 were extracted using the MP Biomedicals FastDNA™ SPIN Kit for Soil from ~400 mg soil (wet weight) following the respective manufacturer’s instructions. DNA from human (~260 mg stool per sample; JMF-2206-10; JMF-2212-18), mouse (one fecal pellet per sample; JMF-2303-06), cow, and calf stool (~250 mg stool per sample; JMF-2305-08) samples were extracted using the QIAamp Fast DNA Stool Mini Kit following the manufacturer’s instructions. DNA from open ocean surface samples was extracted using the Monarch Genomic DNA Purification kit (New England Biolabs, Ipswich, US) and the QIAwave Blood and Tissue kit (QIAGEN GmbH, Hilden, Germany). Surface water and groundwater samples were extracted using a phenol-chloroform-isoamyl alcohol extraction [16]. Water was filled into sterile canisters at the sampling site, and cells were collected on a Sterivex filter (0.2 μm pore size) until clogging (~2 l for surface water, and ~ 10 l for groundwater). DNA from snap-frozen shallow water marine sponge tissue samples (JMF-2407-25) was extracted using the DNase PowerSoil Pro kit (QIAGEN GmbH, Hilden Germany) following the manufacturer’s instructions. Sponge samples were collected under the permit G21/38062.1 and G38062.1 issued by the Great Barrier Reef Marine Park Authority. Amplification, barcoding, normalization, pooling, and sequencing library preparation for all samples was performed using a two-step PCR protocol described in [17]. PCR mastermixes and primer concentration for both primers, as well as cycling conditions for the V4-EMP primers were previously optimized for this protocol and kept as published [18] after a 3 min initial denaturation at 94°C, 35 cycles of denaturation were performed for 45 s at 94°C, annealing for 60 s at 52°C, and elongation for 90 s at 72°C, before a final elongation for 10 min at 72°C. For the V4-EXT primers (forward/reverse: 5'-GTGYCAGMMGBNKCGGTVA-3'/5'-RGACTAMNVRGGTHTCTAAT-3') after a 3 min initial denaturation at 95°C, 35 cycles of denaturation were performed for 40 s at 95°C, primer annealing for 120 s at 55°C, and elongation for 60 s at 72°C, and a final elongation for 7 min at 72°C. For both primer pairs, first step PCRs were performed in 50 μl reaction volume, with the DreamTaq Green PCR Master Mix (ThermoFisher), 0.25 μmol/l of forward and reverse primers each, and 5 μl DNA template. PCR amplification was also performed on ZymoBIOMIC Microbial Community DNA Standard I (D6306) and II (D6311) using both the V4-EMP and V4-EXT primers, and multiple individually amplified replicates of these mock community samples were barcoded and sequenced using the workflow described below. First step PCR products were then cleaned up and normalized using the SequelPrep Normalization Kit (Invitrogen), and 10 μl of first-step PCR product was added as template to a 50 μl barcoding PCR reaction, again containing the DreamTaq PCR Master Mix (ThermoFisher), with 0.8 μmol l–1 of two unique barcoding primers [17]. The barcoding PCR conditions were initial denaturation at 94°C for 4 min; 7 cycles of denaturation for 30 s at 94°C, annealing for 30 s at 52°C, and elongation for 60 s at 72°C; followed by a final elongation step at 72°C for 7 min. Resulting amplicons were purified, normalized, pooled, library prepped, and amplicon libraries were sequenced on the Illumina MiSeq Platform, using v3 chemistry and 600 cycles (2× 300 bp) as described previously [17].

### Amplicon sequence data analysis

Forward reads were trimmed at 220 nt, and reverse reads were trimmed at 150 nt with expected errors of 2 for barcode and primer sequences, respectively. Amplicon sequence variants (ASVs) were inferred from quality-trimmed reads with DADA2 package version 1.20.0 using the default recommended workflow (https://f1000research.com/articles/5-1492). Chimeras were detected by removeBimeraDenovo function in the DADA2 workflow and removed before further analysis. To assure robust sequence novelty analysis, the absence of further chimeric sequences was confirmed by the usearch package with the -uchime3 denovo workflow [19]. ASVs were classified by the assignTaxonomy function of DADA2 with the SILVA r138.1 database taxonomy and Greengene2 database (release 2024.09) [20], with a confidence threshold of 0.5. The fraction of off-target amplicons (i.e. ASVs classified as chloroplast, mitochondria, and eukaryote) in each sample was quantified before they were removed for all downstream analysis. Decontamination was done by decontam package (1.14.0) [21] with the negative control provided in Table S1. Amplification and sequencing were performed on 1513 samples, and data from a total of 938 samples, each with more than 5000 reads in the V4-EXT and V4-EMP datasets after exclusion of off-target amplicons, were included for analyses. Among these 938 samples, the average number of sequence reads is 28 181, and the median number of reads is 23 839. Alpha diversity was summarized by the function amp_alphadiv using the ampvis2 package with rarefy = 5000. Linear regression analysis was performed using the lm() function in R 4.1.2 [22]. To test for statistically significant differences between datasets, paired Wilcoxon tests were performed with the wilcox.test() function with R 4.1.2 [22]. Sequence novelty analysis was performed by blasting ASV sequences against the SILVA database r138.2 and Greengene2 database (backbone and full) with evalue = 1e-5 (Tables S2, S3, and  S4). Hits with identity ≥99 or ≥94.5 and hit length > 200 bp were used for the ASV novelty analysis.

### Digital PCR protocols and absolute 16S rRNA gene copy number determination

Digital PCR assays were performed on a QIAcuity Digital PCR System, using the EvaGreen (EG) PCR Kit, and dilutions of the same DNA extracts used for 16S rRNA gene amplicon sequencing. For the V4-EMP primers, a concentration of 0.4 μM per primer per reaction was used, and dPCR cycling conditions were as follows: 2 min initial denaturation at 95°C, followed by 40 cycles of 30 s/95°C denaturation, 30 s/52°C annealing and 30 s/72°C elongation, and a final 5 min incubation at 40°C were performed. For the V4-EXT primers, a concentration of 1.75 μM per primer per reaction was used, and dPCR cycling conditions were as follows: 2 min initial denaturation at 95°C, followed by 40 cycles of 30 s/95°C denaturation, 45 s/55°C annealing and 30 s/72°C elongation, and a final 5 min incubation at 40°C were performed. A higher concentration of V4-EXT primer was used because during dPCR, the PCR reaction gets partitioned out into nanowells, each containing single DNA template molecules and low reagent amounts. Very high degeneracy primers need to be supplied in higher concentration, so that a primer version with not too many mismatches to any template is present in each nanowell, otherwise gene copy numbers will be underestimated. Fluorescence was read out after each cycle elongation step in both assays. The dPCR data were normalized to 16S rRNA gene copy numbers per μl DNA extract, as collected samples volumes were not sufficiently accurately recorded to perform a biomass-based normalization for the selected, patescibacteria-rich samples.

## Results

### Primer modifications enable expanded *in silico* coverage of the archaeal and bacterial 16S rRNA gene V4 region

We evaluated the *in silico* coverage of three V4 primer sets against bacterial and archaeal sequences, using the most recent release of the SILVA database (r138.2 SSURef NR99) [23] as a reference. We compared without mismatch and one mismatch coverage for the V4 primer pair 515F [10] and 806R [11] used by the Earth Microbiome Project [1] (V4-EMP), the V4 modified primer pair optimized for Patescibacteria, previously referred to as Candidate Phyla Radiation [12] (V4-CPR), and the V4 primer pair further modified for extended taxonomic coverage, which was developed in this study (V4-EXT).

Our analysis showed that V4-EMP primers provided robust *in silico* coverage of most bacterial and archaeal lineages but only captured 19.6% (69.6% with one mismatch) of Patescibacteria and only 56.7% (89.5% with one mismatch) of Chloroflexi sequences (Table S5). The V4-CPR primers achieved an 88.9% (98.5% with one mismatch) and 76.0% (95.2% with one mismatch) *in silico* coverage of Patescibacteria and Chloroflexi 16S rRNA genes, respectively. However, they exhibited limited without mismatch coverage across most archaeal phyla (Table S5). The V4-EXT primers offered a more balanced performance, improving coverage across all bacterial and archaeal phyla, compared to the V4-EMP and V4-CPR primers (Table S5). Notably, the V4-EXT primers significantly improved the *in silico* coverage of archaeal groups poorly covered by the V4-CPR primer, such as the Iainarchaeota and Altiarchaeota phyla (Table S5). Because chloroplast, mitochondrial, and eukaryotic nuclear rRNA genes share partial homology with bacterial and archaeal 16S rRNA genes, particularly in conserved regions that can serve as universal primer binding sites, we explicitly evaluated coverage of these non-target sequences. No significantly increased off-target coverage of chloroplast and eukaryotic rRNA genes was observed with V4-EXT primers, but the fraction of mitochondrial 16 rRNA genes targeted without mismatch was significantly elevated (Table S5).

### *In situ* evaluation of V4-EXT primers across various environmental samples

We experimentally evaluated and compared the performance of the V4-EMP and V4-EXT primers. We generated and analyzed amplicon datasets from a total of 938 samples collected from 4 distinct habitats, including 361 soil samples, 155 samples from ground and freshwater, 9 samples from sponges, 160 samples from surface ocean water, and 253 fecal samples from humans, mice, and cows. These samples originated from 12 independent studies, with sample collection and DNA extraction methods selected according to sample type (see Table S1; see Methods for further details). Alongside samples, we amplified and sequenced two widely used commercial mock community standard (ZymoBIOMICS Microbial Community DNA Standard I and Standard II (Log distribution) with both the V4-EMP and V4-EXT primers.

No significant difference in the recovered relative abundance of mock community members between amplicons generated with the two primer pair versions was detected (Fig. S1). Co-amplification of eukaryotic 18S rRNA gene V4 regions was only detectable in soil and freshwater samples at negligible frequencies with both primer pairs (Table S6). Co-amplification of chloroplast 16S rRNA gene sequences depended on the environment and differed significantly between datasets. As predicted based on *in silico* primer coverage, the proportions of co-amplified chloroplast 16S rRNA genes were similar between primer pairs and were most pronounced in ocean samples, where 15.71% and 17.66% of reads were classified as chloroplast in datasets generated with the V4-EMP and V4-EXT primers, respectively (Table S6). In freshwater samples, 6.29% and 5.14% of reads were identified as chloroplast-derived in the V4-EMP and V4-EXT datasets, respectively. Chloroplast-derived reads were also detected in soil samples but at much lower levels (1%–1.5%), and were nearly absent in gut samples (0.02%) with both primer pairs (Table S6). In contrast, mitochondrial 16S rRNA gene sequences were consistently co-amplified at a significantly higher proportion with the V4-EXT primers (Table S6). In soil samples, the V4-EXT primers resulted in 8.34% of reads being classified as mitochondrial 16S rRNA genes, nearly three times the proportion obtained with the V4-EMP primers (2.83%). A similar pattern was observed in ocean surface water and freshwater samples, where the V4-EXT primers yielded three to five times more mitochondrial reads. In fecal samples, mitochondrial sequences were detected at low levels with both primer pairs (<0.1%) (Table S6). For all downstream analyses, reads classified as chloroplast, mitochondrial, eukaryotic, or unclassified at the phylum level were removed from the datasets.

**Figure 1:**
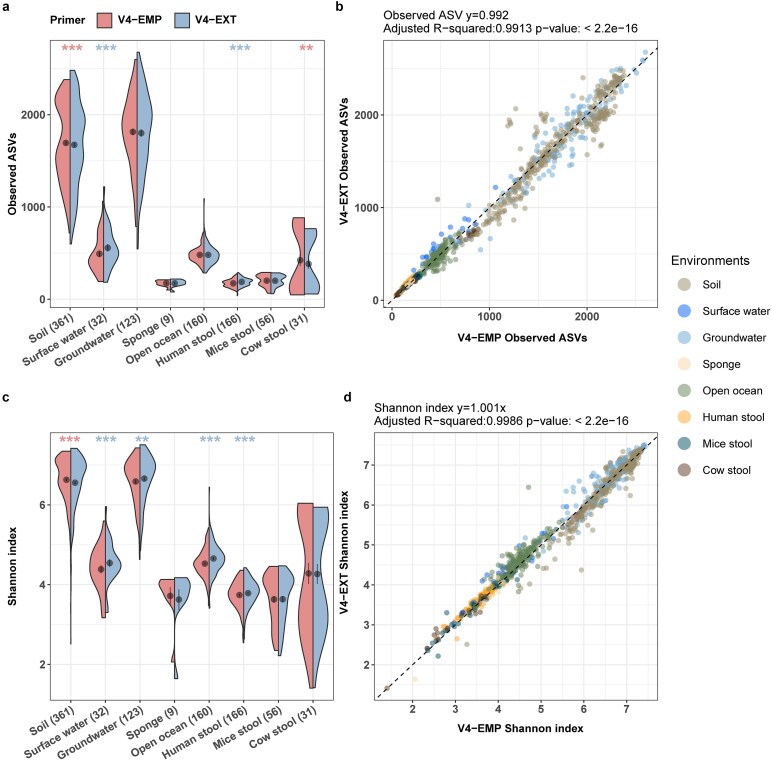
Comparison of (a) observed ASV counts and (b) Shannon index in different datasets generated using the V4-EMP (red) and V4-EXT (blue) primers. The number of samples (in parentheses) and the source environment for each dataset are shown on the *x*-axis. Points and error bars, respectively, represent the mean and standard error (SE) for each dataset. Statistical significance of differences between the datasets was tested by paired Wilcoxon signed-rank tests. ^*^: *P* < .05; ^**^: *P* < .01; ^***^: *P* < .001. The significance indicators are colored in red if the V4-EMP primer dataset has a higher median value and in blue if the V4-EXT primer dataset has a higher median value. Regression analysis of (c) observed ASV counts and (d) Shannon index values between datasets generated using the V4-EMP and the V4-EXT primer pairs. The correlation coefficient, R^2^ value, and *P*-value are indicated in the title of each panel. The theoretical 1:1 line is indicated as a dashed line.

For comparisons of alpha diversity (richness and evenness), each sample in both the V4-EMP and V4-EXT datasets was rarefied to 5000 observations. Significantly higher observed ASV counts and Shannon diversity were observed in the V4-EMP dataset from soil samples (Fig. 1a and b). In contrast, the V4-EXT primers resulted in significantly higher observed ASV counts and elevated Shannon diversity (Fig. 1a and b) in freshwater and groundwater datasets, while only Shannon diversity was found to be significantly higher when using the V4-EMP primers in the open ocean surface water dataset (Fig. 1b). The V4-EXT primers detected a significantly higher number of observed ASVs in human stool samples, whereas they detected significantly fewer ASVs than the V4-EMP in cow stool samples (Fig. 1a). Alpha diversity metrics did not differ significantly when the two primer pairs were applied to mouse stool samples (Fig. 1a and b). Overall, regression analyses across all samples showed that comparable alpha diversity was recovered by the two primer pairs based on both observed ASV counts and the Shannon diversity index (Fig. 1c and d).

### Relative abundance difference at phylum level across different environments

**Figure 2:**
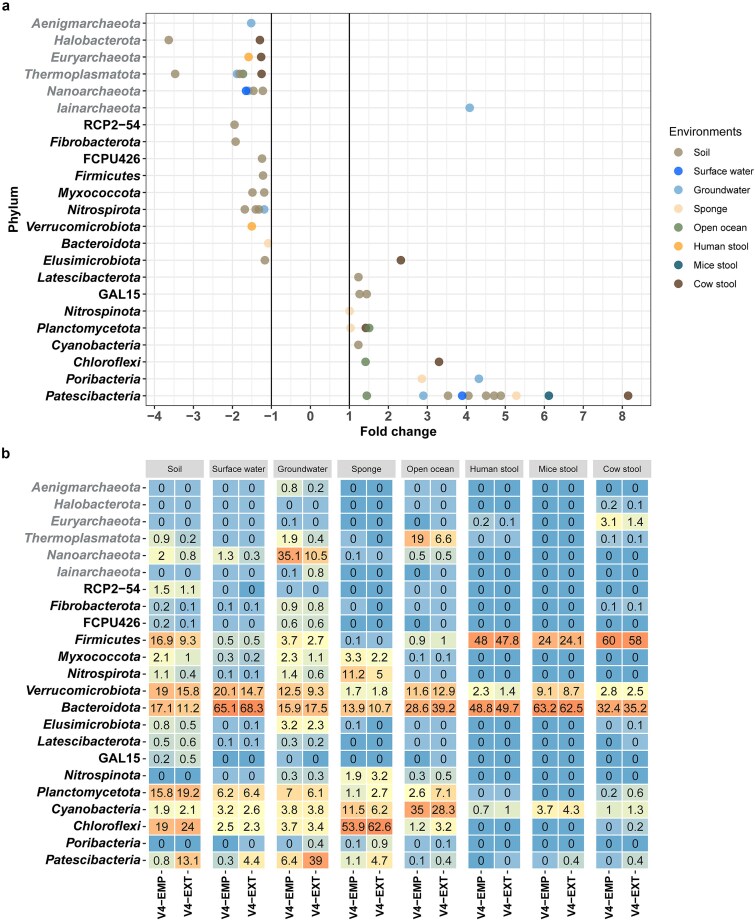
(a) Fold changes in the relative abundances of each phylum in datasets colored by source environment. Each dot represents a research project, and dots are colored by different source habitats. Phyla with a mean relative abundance of >0.1% for which mean relative abundance differences between datasets were above twofold are depicted. Fold change >1 indicates the phylum has a higher mean relative abundance in the V4-EXT dataset and fold change <−1 indicates the phylum has a higher mean relative abundance in the V4-EMP dataset. (b) Phyla-level average relative abundance heatmap across different types of samples grouped by source environment, generated using the V4-EXT versus the V4-EMP primers. Phyla with a mean relative abundance of >0.1% in either dataset are presented. Archaeal phyla are shown in gray font, and bacteria phyla are shown in black font.

Next, we compared phylum-level differential abundance patterns between datasets generated by the two primer pairs. When using the V4-EXT primers, the relative abundance of Patescibacteria was more than twice as high in all sample types except human stool samples, where their average abundance was below 0.1% (Fig. 2a and b, Table S7). For example, Patescibacteria accounted for on average 39% of all reads in groundwater samples from the V4-EXT dataset, but only for 6.4% in the V4-EMP dataset. In surface freshwater samples, Patescibacteria were less dominant, but the V4-EXT primers detected 4.4% reads while the V4-EMP primer detected only 0.3%. In soil samples, the V4-EXT dataset contained on average 13.1% Paterscibacteria reads, while the V4-EMP dataset contained only 0.8% (Fig. 2b, Table S7). Interestingly, in ocean surface water samples, where Patescibacteria were rare regardless of primer choice, Bacterodiota were over 10% more abundant in the V4-EXT dataset (39.2%) than the V4-EMP dataset (28.6%) (Fig. 2b, Table S7). Similarly, Planctomycetota accounted for 7% in V4-EXT datasets from ocean surface samples, but only for 2.6% in the matching V4-EMP dataset. Furthermore, Chloroflexi were generally more abundant in the V4-EXT datasets, especially in ocean, soil, and sponge samples. The V4-EXT primers additionally resulted in significantly higher relative abundances detected for several other phyla in specific environments (Fig. 2a, Table S7): Poribacteria and Iainarchaeota in groundwater samples; Cyanobacteria, Latescibacterota, and GAL15 in soil samples; Planctomycetota and Chloroflexi in ocean surface water samples; and Planctomycetota, Elusimicrobiota, and Chloroflexi in cow stool samples. Conversely, the V4-EMP datasets contained statistically significantly higher relative abundances of five archaeal phyla (Thermoplasmatota, Nanoarchaeota, Halobacterota, Euryarchaeota, and Aenigmarchaeota) across all sample types (Fig. 2a, Table S7). Among these, Thermoplasmatota were particularly abundant (19%) in the open ocean surface water sample, and Nanoarchaeota (35.1%) in the groundwater sample datasets generated with V4-EMP primers, but accounted for only 6.5% and 10.5%, respectively, in V4-EXT datasets (Fig. 2a, Table S7). The remaining archaeal phyla were comparatively rare in both datasets from the samples analyzed in this study. Furthermore, seven bacterial phyla (Elusimicrobiota, Nitrospirota, Myxococcota, Firmicutes, FCPU426, Fibrobacterota, and RCP2-54) were relatively more abundant in the V4-EMP soil datasets, while one phylum was more abundant in the V4-EMP dataset from groundwater (Nitrospirota), human stool (Verrucomicrobiota), and sponge (Bacteroidota) samples, respectively. Although not being statistically significantly different, Firmicutes-related sequences were notably more abundant in the V4-EMP (16.9%) than the V4-EXT (9.3%) soil dataset, while Cyanobacteria and Nitrospirota related reads were double as abundant in the V4-EMP dataset (11.5% and 11.2%) than the V4-EXT dataset (6.2% and 5.0%) from sponge samples.

As the relative abundance of Patescibacteria was significantly higher in all V4-EXT sample types except human stool samples, pronounced changes in their relative abundances could have masked or inflated shifts in the relative abundance of other phyla. To better evaluate the effect of primer choice on the residual community, we compared the relative abundances of phyla between datasets after removing reads classified as Patescibacteria. As expected, the differences in the relative abundance of other phyla between the V4-EXT and V4-EMP datasets from soil and groundwater samples became less pronounced, as Patescibacteria were highly abundant in the V4-EXT datasets from these samples (Fig. S2A and B, Table S7). In groundwater samples, the relative abundance of archaea increased, with 22.4% and 38.4% of reads classified as Nanoarchaeota in the V4-EXT and V4-EMP datasets, respectively. Yet, Nanoarchaeota and other rare archaeal phyla still had a higher relative abundance in V4-EMP datasets. However, the relative abundance of Iainarchaeota was approximately four times higher in the V4-EXT dataset (Fig. S2A). While these results might suggest a potential primer bias affecting specific archaeal phyla, the overall low relative abundance of these phyla, even in the V4-EMP groundwater dataset, must be noted. Such low relative abundance may exaggerate differences detected in differential abundance analyses (Fig. S3), as the differentials are more strongly affected by the improved detection and thus increased relative abundance of other taxa. Notably, the relative abundance of numerous bacterial phyla, including Bacteroidota, Planctomycetota, Chloroflexi, and Cyanobacteria, was correspondingly substantially higher in the V4-EXT dataset. A similar trend was observed in soil samples after excluding Patescibacteria reads, with a higher relative abundance of Planctomycetota and Chloroflexi in the V4-EXT dataset, while Verrucomicrobiota, Bacteroidota, and Firmicutes remained relatively more abundant in the V4-EMP dataset (Fig. S2).

### Comparison of absolute 16S rRNA gene copy number profiles generated by V4-EXT and V4-EMP

To compare differences in absolute 16S rRNA gene copy numbers obtained using the two primer pairs, we selected 138 (out of 938; 15%) samples, from groundwater and freshwater, for digital PCR (dPCR) based quantification of 16S rRNA gene copy numbers. The average normalized 16S rRNA gene copy numbers obtained by dPCR with the V4-EMP primer pair were 1.48-fold (median) higher than those obtained with the V4-EXT primer pair (Fig. S4A, Wilcoxon paired test, *P* = 7.02 × 10^−13^). This can be attributed to the much higher degeneracy of the V4-EXT primer pair, which impacts the suitability of this primer pair for quantitative PCR. The primer concentrations in the dPCR assay with V4-EXT are in consequence 4-fold higher than in the V4-EMP assay, and yet do not entirely compensate for the high degeneracy.

We then compared the absolute 16S rRNA gene copy numbers for each phylum across all groundwater and surface water samples (Fig. S4B). Patescibacteria exhibited orders of magnitude higher absolute 16S rRNA gene copy numbers in the V4-EXT dataset compared to the V4-EMP dataset (surface water: V4-EXT mean = 77 960 copies/μl, V4-EMP mean = 5535 copies/μl; groundwater: V4-EXT mean = 248 228 copies/μl, V4-EMP mean = 83 860 copies/μl). Similarly, Poribacteria and Iainarchaeota were significantly more abundant in the V4-EXT datasets, whereas several other archaeal phyla were detected with higher absolute gene copy numbers with the V4-EMP primer pair in these ecosystems.

### More novel ASVs are observed in V4-EXT datasets

Interpreting 16S rRNA gene amplicon data relies heavily on databases that provide the necessary taxonomic, functional, and ecological context for ASVs or operational taxonomic units (OTUs) generated from raw sequences. ASVs and OTUs without close relatives in these databases likely represent novel biological entities. Thus, we focused specifically on such novel sequence variants generated by the V4-EMP and V4-EXT primer pairs.

**Figure 3:**
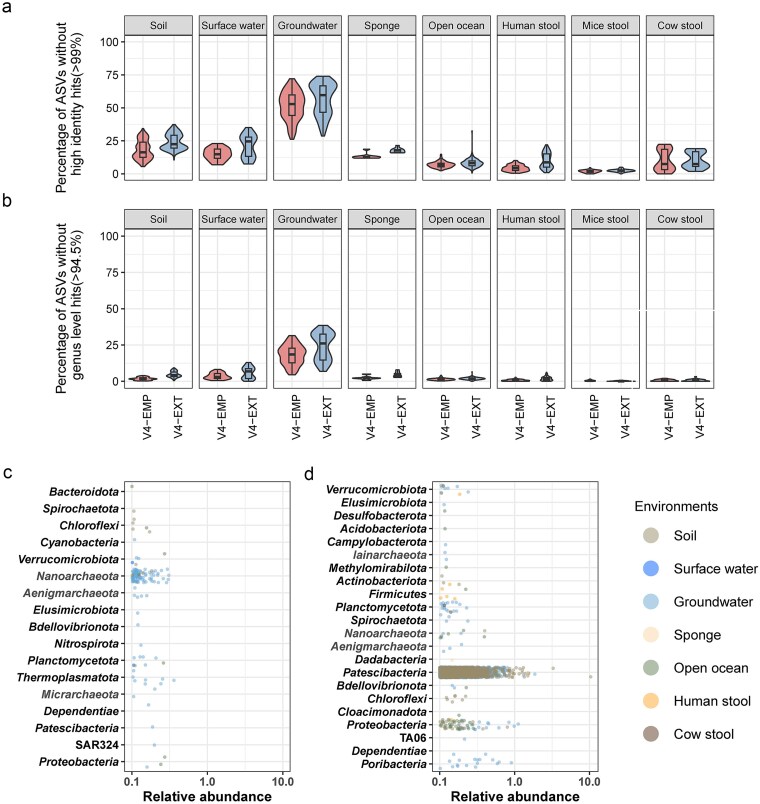
Comparison of ASV novelty generated by the V4-EMP (red) and V4-EXT (blue) primer pairs. The percentage of ASVs in each sample type (a) without high-identity (>99%) matches and (b) without genus-level identity (>94.5%) matches to the SILVA r138.2 database. The phylum-level classification of ASVs without genus-level matches in the SILVA v138.2 database in (c) the V4-EMP and (d) the V4-EXT dataset. Each dot represents an ASV in a sample, and only ASVs with >0.1% relative abundance are shown. Relative abundance is displayed on the *x*-axis, on a logarithmic scale. Different sample types are depicted by dots of different colors. The phylum SAR324 clade (marine group B) is indicated by SAR324. Archaeal phyla are shown in gray font, and bacterial phyla are shown in black font.

First, we compared the fraction of novel ASVs, lacking high-identity matches (<99% identity) [24] or genus-level matches (<94.5% identity) [25] in the SILVA database. The V4-EXT primers detected significantly more novel ASVs at both thresholds across all sample types except cow stool samples, for which both primer pairs yielded similar proportions of novel ASVs (Fig. 3a). Groundwater samples exhibited the highest proportion of novel ASVs with both primer pairs. Nearly half of the ASVs obtained from groundwater samples by the V4-EXT primers (56.5% ± 12.0%, mean ± SD) lacked high-identity matches in the SILVA database, and 23.4% ± 9.3% of these ASVs could not be assigned to a genus. Using the V4-EMP primers on the same samples yielded 51.5 ± 10.7% ASVs without high-identity matches in the SILVA database (Fig. 3a and b). As expected, stool samples from humans, mice, and cows showed the lowest levels of novelty (on average 4.6% ± 4.1% and 8.4% ± 6.0% in the V4-EMP and V4-EXT datasets, respectively), confirming that these microbial communities are comparatively well characterized. Across all samples, cumulatively, 33.0% of ASVs from the V4-EXT dataset and 30.7% from the V4-EMP dataset lacked high-identity matches. At the genus level, 11.6% (V4-EXT) and 8.6% (V4-EMP) of ASVs could not be assigned across all samples cumulatively.

To quantify unique novelty detected by either primer pair, we further examined the ASVs lacking genus-level matches in the SILVA database that were only detected in the V4-EMP or the V4-EXT datasets. We found 56 278 ASVs with a relative abundance >0.01% without a genus-level match in the SILVA database unique to the V4-EXT dataset, while the V4-EMP dataset contained only 14 744 unique genus-level novel ASVs (Fig. S5). Nanoarchaeota ASVs comprise 63.5% (*n* = 9369) of these novel ASVs in the V4-EMP dataset, followed by Patescibacteria (*n* = 976, 6.6%), Proteobacteria (*n* = 727, 4.9%), and Verrucomicrobiota (*n* = 678, 4.6%) ASVs. In the V4-EXT datasets, 88.4% (*n* = 49 754) of the genus-level novel ASVs were classified as Patescibacteria, 2.2% (*n* = 1260) as Verrucomicrobiota, 2.0% (*n* = 1137) as Proteobacteria, 1.4% (*n* = 783) as Iainarchaeota, and 0.7% (*n* = 446) as Nanoarchaeota (Fig. S5). The overwhelming majority of genus-level novel ASVs in both datasets (98.6% and 99.3% in V4-EXT and V4-EMP datasets, respectively) were extremely rare and displayed a cumulative relative abundance <0.1%.

Notably, among the ASVs without genus-level matches and with a relative abundance above 0.1%, only 2% ASVs (*n* = 110) were unique to the V4-EMP datasets, most of which were classified as Nanoarchaeota (*n* = 74, 67.3% of novel ASVs). They primarily originated from groundwater samples and showed individual relative abundances <0.3% (Fig. 3c). The V4-EMP primer pair also uniquely detected 29 novel genus-level bacterial ASVs, which were distributed among 13 different phyla (Fig. 3c). The V4-EXT primer pair detected 7 times more genus-level unassigned ASVs (*n* = 792). The majority were identified as Patescibacteria (*n* = 704, 88.9%), and originated from soil and groundwater ecosystems, highlighting substantial undiscovered diversity within this bacterial phylum, not captured by the V4-EMP primers. Additionally, we identified numerous novel, relatively highly abundant ASVs belonging to the order Rickettsiales (Alphaproteobacteria), detected across freshwater, soil, and ocean water column samples, as well as novel Poribacteria ASVs from freshwater environments (Fig. 3d). We further compared ASVs which were only detected in the V4-EMP datasets (*n* = 20 120) and the V4-EXT (*n* = 20 875) datasets, corresponding to 4.62% and 7.28% cumulative abundance, respectively. Most of the unique ASVs in both datasets were of extremely low relative abundance, accounting for a median of 0.016% and 0.012% in the V4-EXT and V4-EMP datasets, respectively (Fig. S6A). Among unique ASVs with >0.1% relative abundance, most (164 out of 560; 29.3%) were classified as Patescibacteria and only detected in the V4-EXT datasets (Fig. S6C).

## Discussion

Here, we present an additional modification to a recently published primer pair [9] targeting the V4 region of bacterial and archaeal 16S rRNA genes (V4-EXT), with an even greater overall *in silico* coverage. We experimentally compared the performance of the V4-EXT primer pair to the V4-EMP primers, which are the most commonly used 16S rRNA gene-targeted primers across many fields of ecological research [12] using sequence datasets generated with both primer pairs from 938 samples from different ecosystems.

*In silico* analysis showed that the V4-EXT primer pair should particularly improve the ability to detect Patescibacteria, Chloroflexi, and Iainarchaeota. Consistently, in empirical datasets, the relative abundance of Patescibacteria was significantly higher across all sample types in V4-EXT datasets, except in human stool samples, in which these bacteria are nearly absent (Fig. 2). Also, marked genus and species novelty among the Patescibacteria was detected when V4-EXT primers were applied (Fig. 3). Significant and drastic differences in community composition data between datasets were detected particularly in high richness, high diversity samples from soil and groundwater environments.

It has to be acknowledged that both the V4-EMP and V4-EMP datasets generated and compared here remain affected by distinct primer, and shared PCR and amplicon sequencing biases. In environmental studies, metagenomic sequencing can thus serve as a complementary approach for community profiling as it does not rely on PCR amplification. However, direct comparison between amplicon- and metagenomics-derived abundance profiles is not straightforward, as metagenome-derived community composition profiles are affected by a distinct set of systematic biases. Yet, results from several recent metagenomic surveys support our findings that groundwater and soils are major reservoirs of untapped prokaryotic diversity, especially regarding Patescibacteria and the archaeal DPANN superphylum [13, 26]. In groundwater samples, where Patescibacteria accounted for on average >35% of all amplicon sequences in V4-EXT datasets, their relative abundance is severely underestimated with the V4-EMP primers (6.4%). The V4-EXT results align closer with observations from groundwater metagenomes, where Patescibactria are reported to be the dominant phylum with relative abundances ranging from 10%–40% [13, 27], and are increasingly recognized as abundant core community members. Patescibacteria are characterized by ultra-small cells and streamlined genomes with severely reduced biosynthetic and regulatory capacities, suggesting strong reliance on external sources for metabolites and interactions with partner organisms [28, 29]. Studies indicate that specific Patescibacteria co-occur with specific other bacterial or archaeal taxa, forming symbiotic interactions, usually as parasitic epibionts [30, 31].

Similarly, members of the archaeal superphylum DPANN, which among others includes the phyla Nanoarchaeota, Aenigmarchaeota, Woesearchaeota, Micrarchaeota, and Iainarchaeota, are small cells with small genomes and limited metabolic capacities [30]. Like the Patescibacteria, many DPANN archaea are obligate episymbionts or parasites, physically attaching to host cells, such as Thermoproteota [31]. Metagenomic data suggest they can play a role in carbon, sulfur, and nitrogen cycling, though the extent of their involvement and influence on these elemental cycles remains poorly constrained [32]. The V4-EXT primers showed a significantly and markedly improved coverage of Iainarchaeota (Table S5), resulting in their significantly improved detection in groundwater samples (Fig. 2). Small, enigmatic archaeal cells [32] have been shown to comprise up to 25% of groundwater microbial communities, with relative abundances of individual phyla being highly dynamic and patchy [13]. Thus, an inclusive detection of all DPANN phyla is important for accurate interpretation of their environmental significance and distribution. However, it is important to acknowledge that in our dataset, Nanoarchaeota still manyfold outnumber Iainarchaeota (Fig. 2), and despite equal *in silico* coverage, the V4-EXT primer detected a significantly lower relative abundance and sequence novelty within the Nanoarchaeota than the V4-EMP primers (Fig. 2, Fig. S2).

When the compositional 16S rRNA gene amplicon sequencing results are normalized by dPCR and converted to absolute 16S rRNA gene copy numbers, the observations remain the same: the V4-EXT dataset provides a markedly improved detection of Patescibacteria and Iainarchaeota, but results in the detection of lower numbers of Nanoarchaeota, and several other, less abundant archaeal phyla, despite equivalent *in silico* coverage (Fig. S3). We were in this study not able to resolve if this discrepancy between *in silico* coverage and experimental detection of Nanoarchaeota is due to a real phylum-specific amplification bias, or is based on the overall poorer performance of the highly degenerate V4-EXT primers in dPCR assays. In future experiments, DNA or whole cell spike-in based normalization of 16S rRNA gene abundance could be used to further investigate this discrepancy between *in silico* prediction and *in situ* results.

We also found that the V4-EXT primers more effectively detected Poribacteria and Chloroflexi in sponge samples. These results align with global surveys of sponge microbiomes, conducted with a V3-V4 region-targeted 16S rRNA primer pair, which showed that both Chloroflexi and Poribacteria are enriched in marine sponges [33]. In metagenomic datasets from the Red Sea sponge Hyrtios erectus, Poribacteria accounted for ~7% and Chloroflexi for 10.7% of the microbial community [34]. In a deep-sea sponge study, Poribacteria and Chloroflexi were identified to be among the most transcriptionally active phyla, contributing 17% and 12% of microbial transcripts, respectively, second only to Proteobacteria (23.7%) [35]. Beyond sponges, Chloroflexi are also known to be abundant in some soils and sediments, representing 9%–16% of the bacterial community in, for example, river sediment samples from Rifle, Colorado [18], and reaching up to 60% relative abundance in deep marine sediments [36] and global soil diversity surveys [26].

Notably, several, mainly rare (<1% relative abundance) bacterial phyla were found to be statistically significantly less abundant in datasets generated with the V4-EXT (Fig. S2), despite equal or better *in silico* coverage of the corresponding phyla (Table S5). These shifts are more likely observed due to the improved detection and related increased abundance of other taxa, rather than due to primer biases as illustrated by the minimal unique sequence novelty detected in the V4-EMP dataset (Fig. 3) and the comparable absolute 16S rRNA gene copy numbers detected for bacterial phyla in dPCR assays with both primers (Fig. S3).

While no difference in co-amplification of chloroplast 16S rRNA gene and eukaryotic 18S rRNA gene sequences was observed between primer pairs across sample types, mitochondrial 16S rRNA gene sequences were more strongly affected by primer choice, with the V4-EXT primers resulting in approximately three times more mitochondrial reads (Table S6). Still, the highest proportion, observed in soil samples, was only about 10% and is not high enough to prohibit the use of V4-EXT in any of the here tested source environment types, including directly host-derived samples (sponge and fecal samples).

In summary, we here present the V4-EXT primer pair: an improved successor to V4-CPR [12] and revision of the well-established and widely used V4-EMP primers [10, 11], with equal or improved in silico coverage across all bacterial and archaeal lineages. We furthermore demonstrate using a large and diverse collection of samples that the application of V4-EXT has no detrimental drawbacks, but drastically alters microbial community compositions recovered from various environmental habitats, where previously poorly covered taxa like the Patescibacteria can account for up to on average 35% of all reads.

Importantly, it has to be kept in mind that due to their high level of degeneracy, the V4-EXT primers are not optimally suited for 16S rRNA gene copy number determination based on quantitative PCR assays, and can result in systematically slightly lower 16S rRNA gene copy number quantifications than the V4-EMP primers.

## Supplementary Material

Supplementary_figures_ycag141

Supplementary_tables_ycag141

## Data Availability

The 16S rRNA gene amplicon sequence data and associated metadata are available in the NCBI Sequences Read Archive (SRA) under the following BioProject IDs: PRJNA1366826 (JMF-2206-10), PRJNA1363873 (JMF-2210-08), PRJNA1366128 (JMF-2212-18), PRJNA1363863 (JMF-2212-20), PRJNA1226173 (JMF-2303-06), PRJNA1366369 (JMF-2305-08), PRJNA1366129 (JMF-2301-09), PRJNA1366130 (JMF-2302-05), PRJNA1367499 (JMF-2311-06), PRJNA1366290 (JMF-2401-02), PRJNA1366145 (JMF-2401-04), PRJNA1366146 (JMF-2401-05), PRJNA1367501/PRJNA1366385 (JMF-2407-25), PRJNA595173 (negative controls).

## References

[1] Thompson LR, Sanders JG, McDonald D. et al. A communal catalogue reveals Earth’s multiscale microbial diversity. *Nature* 2017;551:457–63. 10.1038/nature24621 29088705 PMC6192678

[2] Wu L, Ning D, Zhang B. et al. Global diversity and biogeography of bacterial communities in wastewater treatment plants. *Nat Microbiol* 2019;4:1183–95. 10.1038/s41564-019-0426-5 31086312

[3] Alteio LV, Séneca J, Canarini A. et al. A critical perspective on interpreting amplicon sequencing data in soil ecological research. *Soil Biol Biochem* 2021;160:108357. 10.1016/j.soilbio.2021.108357

[4] Seki D, Mayer M, Hausmann B. et al. Aberrant gut-microbiota-immune-brain axis development in premature neonates with brain damage. *Cell Host Microbe* 2021;29:1558–1572.e6. 10.1016/j.chom.2021.08.004 34480872 PMC8525911

[5] Wu L, Zhang Y, Guo X. et al. Reduction of microbial diversity in grassland soil is driven by long-term climate warming. *Nat Microbiol* 2022;7:1054–62. 10.1038/s41564-022-01147-3 35697795

[6] Zhang L, Yin W, Wang C. et al. Untangling microbiota diversity and assembly patterns in the world’s largest water diversion canal. *Water Res* 2021;204:117617. 10.1016/j.watres.2021.117617 34555587

[7] Lee CK, Herbold CW, Polson SW. et al. Groundtruthing next-gen sequencing for microbial ecology–biases and errors in community structure estimates from PCR amplicon pyrosequencing. *PloS One* 2012;7:e44224. 10.1371/journal.pone.0044224 22970184 PMC3435322

[8] Schirmer M, Ijaz UZ, D’Amore R. et al. Insight into biases and sequencing errors for amplicon sequencing with the Illumina MiSeq platform. *Nucleic Acids Res* 2015;43:e37. 10.1093/nar/gku1341 25586220 PMC4381044

[9] Qin Y, Wu L, Zhang Q. et al. Effects of error, chimera, bias, and GC content on the accuracy of amplicon sequencing. *Msystems* 2023;8:e01025–3. 38038441 10.1128/msystems.01025-23 PMC10734440

[10] Parada AE, Needham DM, Fuhrman JA. Every base matters: assessing small subunit rRNA primers for marine microbiomes with mock communities, time series and global field samples. *Environ Microbiol* 2016;18:1403–14. 10.1111/1462-2920.13023 26271760

[11] Apprill A, McNally S, Parsons R. et al. Minor revision to V4 region SSU rRNA 806R gene primer greatly increases detection of SAR11 bacterioplankton. *Aquat Microb Ecol* 2015;75:129–37. 10.3354/ame01753

[12] Hu H, Kristensen JM, Herbold CW. et al. Global abundance patterns, diversity, and ecology of Patescibacteria in wastewater treatment plants. *Microbiome* 2024;12:55. 10.1186/s40168-024-01769-1 38493180 PMC10943839

[13] He C, Keren R, Whittaker ML. et al. Genome-resolved metagenomics reveals site-specific diversity of episymbiotic CPR bacteria and DPANN archaea in groundwater ecosystems. *Nat Microbiol* 2021;6:354–65. 10.1038/s41564-020-00840-5 33495623 PMC7906910

[14] Shaiber A, Willis AD., Delmont TO et al. Functional and genetic markers of niche partitioning among enigmatic members of the human oral microbiome. *Genome Biol* 2020;21:292. 10.1186/s13059-020-02195-w 33323122 PMC7739484

[15] Chiriac M-C, Bulzu P-A, Andrei A-S. et al. Ecogenomics sheds light on diverse lifestyle strategies in freshwater CPR. *Microbiome* 2022;10:84. 10.1186/s40168-022-01274-3 35659305 PMC9166423

[16] Lueders T, Manefield M, Friedrich MW. Enhanced sensitivity of DNA- and rRNA-based stable isotope probing by fractionation and quantitative analysis of isopycnic centrifugation gradients. *Environ Microbiol* 2004;6:73–8. 10.1046/j.1462-2920.2003.00536.x 14686943

[17] Pjevac P, Hausmann B, Schwarz J. et al. An economical and flexible dual barcoding, two-step PCR approach for highly multiplexed amplicon sequencing. *Front Microbiol* 2021;12:669776. 10.3389/fmicb.2021.669776 PMC8173057 34093488

[18] Hug LA, Castelle CJ, Wrighton KC. et al. Community genomic analyses constrain the distribution of metabolic traits across the Chloroflexi phylum and indicate roles in sediment carbon cycling. *Microbiome* 2013;1:22. 10.1186/2049-2618-1-22 24450983 PMC3971608

[19] Edgar RC . UCHIME2: improved chimera prediction for amplicon sequencing. 2016;074252. 10.1101/074252

[20] McDonald D, Jiang Y, Balaban M. et al. Greengenes2 unifies microbial data in a single reference tree. *Nat Biotechnol* 2024;42:715–8. 10.1038/s41587-023-01845-1 37500913 PMC10818020

[21] Davis NM, Proctor D, Holmes SP. et al. Simple statistical identification and removal of contaminant sequences in marker-gene and metagenomics data. *bioRxiv* 2017;221499. 10.1101/221499 PMC6298009 30558668

[22] R Core Team . R: A Language and Environment for Statistical Computing. Vienna, Austria: R Foundation for Statistical Computing, 2021.

[23] Yilmaz P, Parfrey LW, Yarza P. et al. The SILVA and “all-species living tree project (LTP)” taxonomic frameworks. *Nucleic Acids Res* 2014;42:D643–8. 10.1093/nar/gkt1209 24293649 PMC3965112

[24] Dueholm MKD, Nierychlo M, Andersen KS. et al. MiDAS 4: a global catalogue of full-length 16S rRNA gene sequences and taxonomy for studies of bacterial communities in wastewater treatment plants. *Nat Commun* 2022;13:1908. 10.1038/s41467-022-29438-7 35393411 PMC8989995

[25] Yarza P, Yilmaz P, Pruesse E. et al. Uniting the classification of cultured and uncultured bacteria and archaea using 16S rRNA gene sequences. *Nat Rev Microbiol* 2014;12:635–45. 10.1038/nrmicro3330 25118885

[26] Bahram M, Hildebrand F, Forslund SK. et al. Structure and function of the global topsoil microbiome. *Nature* 2018;560:233–7. 10.1038/s41586-018-0386-6 30069051

[27] Anantharaman K, Brown CT, Hug LA. et al. Thousands of microbial genomes shed light on interconnected biogeochemical processes in an aquifer system. *Nat Commun* 2016;7:13219. 10.1038/ncomms13219 27774985 PMC5079060

[28] Castelle CJ, Brown CT, Anantharaman K. et al. Biosynthetic capacity, metabolic variety and unusual biology in the CPR and DPANN radiations. *Nat Rev Microbiol* 2018;16:629–45. 10.1038/s41579-018-0076-2 30181663

[29] Tian J, Utter DR, Cen L. et al. Acquisition of the arginine deiminase system benefits epiparasitic Saccharibacteria and their host bacteria in a mammalian niche environment. *Proc Natl Acad Sci* 2022;119:e2114909119. 10.1073/pnas.2114909119 34992141 PMC8764695

[30] Dombrowski N, Lee J-H, Williams TA. et al. Genomic diversity, lifestyles and evolutionary origins of DPANN archaea. *FEMS Microbiol Lett* 2019;366:fnz008. 10.1093/femsle/fnz008 30629179 PMC6349945

[31] Wurch L, Giannone RJ, Belisle BS. et al. Genomics-informed isolation and characterization of a symbiotic Nanoarchaeota system from a terrestrial geothermal environment. *Nat Commun* 2016;7:12115. 10.1038/ncomms12115 27378076 PMC4935971

[32] Vigneron A, Cruaud P, Lovejoy C. et al. Genomic evidence of functional diversity in DPANN archaea, from oxic species to anoxic vampiristic consortia. *ISME Commun* 2022;2:4. 10.1038/s43705-022-00088-6 37938653 PMC9723730

[33] Busch K, Slaby BM, Bach W. et al. Biodiversity, environmental drivers, and sustainability of the global deep-sea sponge microbiome. *Nat Commun* 2022;13:5160. 10.1038/s41467-022-32684-4 36056000 PMC9440067

[34] Samak ME, Solyman SM, Hanora A. et al. Metagenomic mining of two Egyptian Red Sea sponges associated microbial community. *BMC Microbiol* 2024;24:315. 10.1186/s12866-024-03299-0 39192220 PMC11351353

[35] Díez-Vives C, Riesgo A. High compositional and functional similarity in the microbiome of deep-sea sponges. *ISME J* 2024;18:wrad030. 10.1093/ismejo/wrad030 38365260 PMC10837836

[36] Wu J, Wang L, Du J. et al. Biogeographic distribution, ecotype partitioning and controlling factors of Chloroflexi in the sediments of six hadal trenches of the Pacific Ocean. *Sci Total Environ* 2023;880:163323. 10.1016/j.scitotenv.2023.163323 37030385

