# Supplementary material for: Revised 16S rRNA V4 hypervariable region targeting primers enhance detection of Patescibacteria and other lineages across diverse environments
Source: ISME Commun. 2026 May 21;6(1):ycag141. doi: 10.1093/ismeco/ycag141 (PMC13271367; doi:10.1093/ismeco/ycag141)
Supplement: Supplementary_figures_ycag141 [file supplementary_figures_ycag141.docx]

**Supplementary Material for**

**Revised 16S rRNA V4 hypervariable region targeting primers enhance detection of *Patescibacteria* and other lineages across diverse environments**

Huifeng Hu^1,2*^, Clemens Karwautz^3^, Kalina Duszka^4,5^, Thomas Karner^6^, Isabella C. Wagner^1,6^, Christoph Grander^7^, Wilhelm Grander^8^, Laura Steinwidder^9^, Lucilla Boito^9^, Viktor Van de Velde^10,11^, Marijn Bauters^10^, Pascal Boeckx^11^, David Seki^1^, Bettina Glasl^1^, Stefan Thiele^1^, Hannes Schmidt^1^, Joana Séneca^1,12,13^, Michael Wagner^1,13,14^, Petra Pjevac^1,13,15*^

1. Centre for Microbiology and Environmental Systems Science, University of Vienna. Djerassiplatz 1, 1030 Vienna, Austria
2. ​Doctoral School in Microbiology and Environmental Science, University of Vienna. Universitätsring 1, 1010 Vienna, Austria
3. Department of Functional and Evolutionary Ecology, Faculty of Life Sciences, University of Vienna, Djerassiplatz 1, 1030 Vienna, Austria
4. Department of Nutritional Sciences, Faculty of Lifesciences, University of Vienna, Josef-Holaubek-Platz 2, 1090 Vienna, Austria
5. Centre for Animal Nutrition and Welfare, University of Veterinary Medicine, Veterinärplatz 1, 1210 Vienna, Austria
6. Faculty of Psychology, University of Vienna, Liebiggasse 5, 1010 Vienna, Austria
7. Department of Internal Medicine I, Gastroenterology, Hepatology, Endocrinology, and Metabolism, Medical University of Innsbruck, Innrain 52, 6020 Innsbruck, Austria
8. Department of Internal Medicine, Hall State Hospital, Milserstrasse 10, 6060 Hall in Tirol, Austria
9. Biobased Sustainability Engineering (SUSTAIN), Department of Bioscience Engineering, University of Antwerp, Antwerp 2020, Belgium
10. Department of Environment, Faculty of Bioscience Engineering, Ghent University, Coupure Links 653, 9000 Gent, Belgium
11. Department of Green Chemistry and Technology, Faculty of Bioscience Engineering, Ghent University, Coupure Links 653, 9000 Gent, Belgium
12. Department of Laboratory Medicine, Medical University of Vienna. Währinger Gürtel 18-20, 1090 Vienna, Austria
13. Joint Microbiome Facility of the Medical University of Vienna and the University of Vienna. Djerassiplatz 1, 1030 Vienna, Austria
14. Center for Microbial Communities, Department of Chemistry and Bioscience, Aalborg University. Fredrik Bajers Vej 7H, 9220 Aalborg, Denmark
15. Environment and Climate Hub (ECH), University of Vienna, Augasse 2-6, 1090 Vienna, Austria.

*Corresponding authors: Huifeng Hu ([Huifeng.hu@univie.ac.at](mailto:Huifeng.hu@univie.ac.at)) and Petra Pjevac ([petra.pjevac@univie.ac.at](mailto:petra.pjevac@univie.ac.at)). Mailing address: Centre for Microbiology and Environmental Systems Science, University of Vienna. Djerassiplatz 1, 1030 Vienna, Austria

Running title: V4-EXT – a revised 16S rRNA primer

**Supplementary figures**


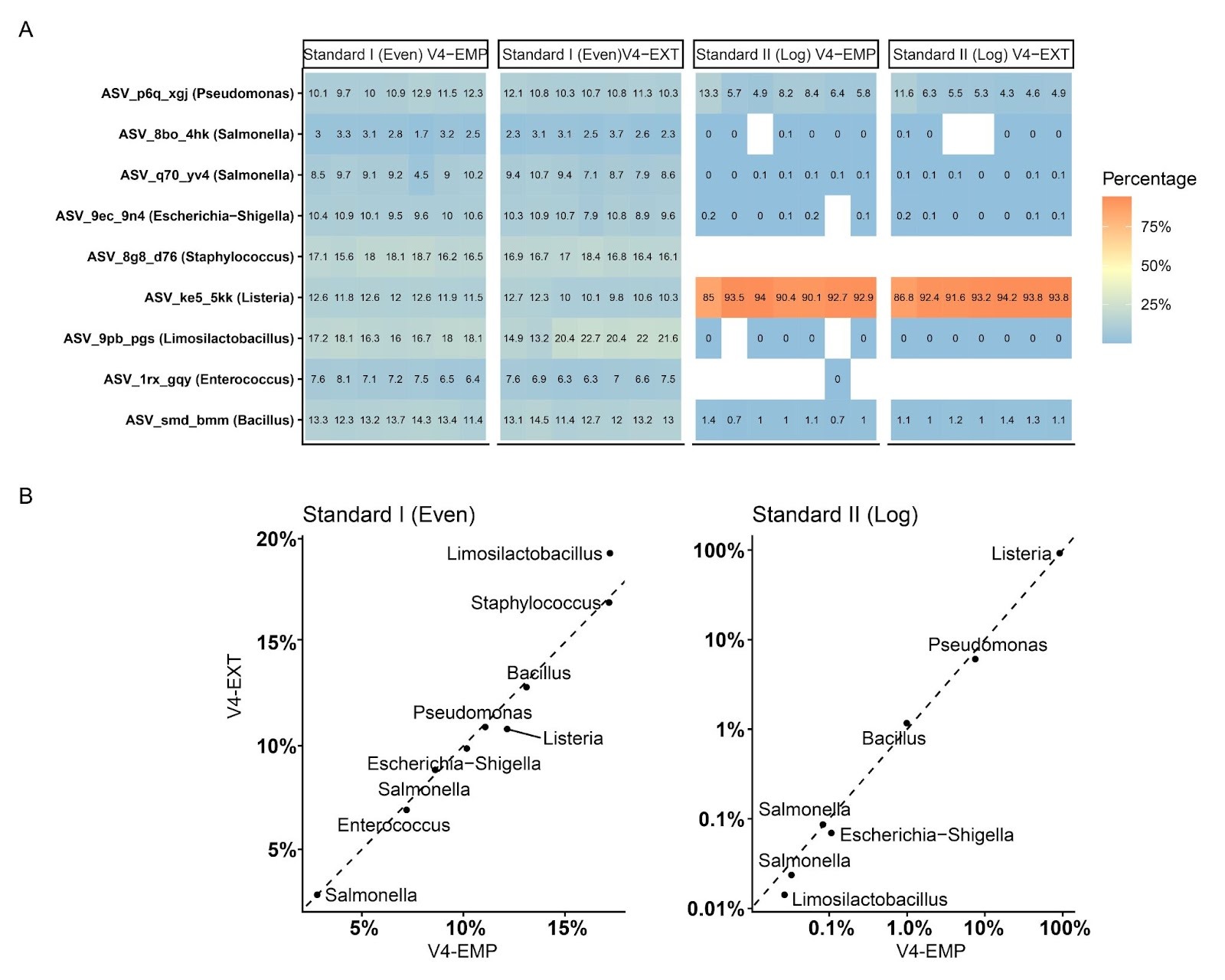


**Figure S1**. A) Per replicate relative abundance of ASVs detected in the ZymoBIOMIC Microbial Community DNA Standard I and IIafter amplification with the V4-EMP and V4-EXT primers. No significant differences in ASV abundances between datasets were detected. B) Regression analysis of average mock community ASV relative abundance values between datasets generated using the V4-EMP and the V4-EXT primer pairs.


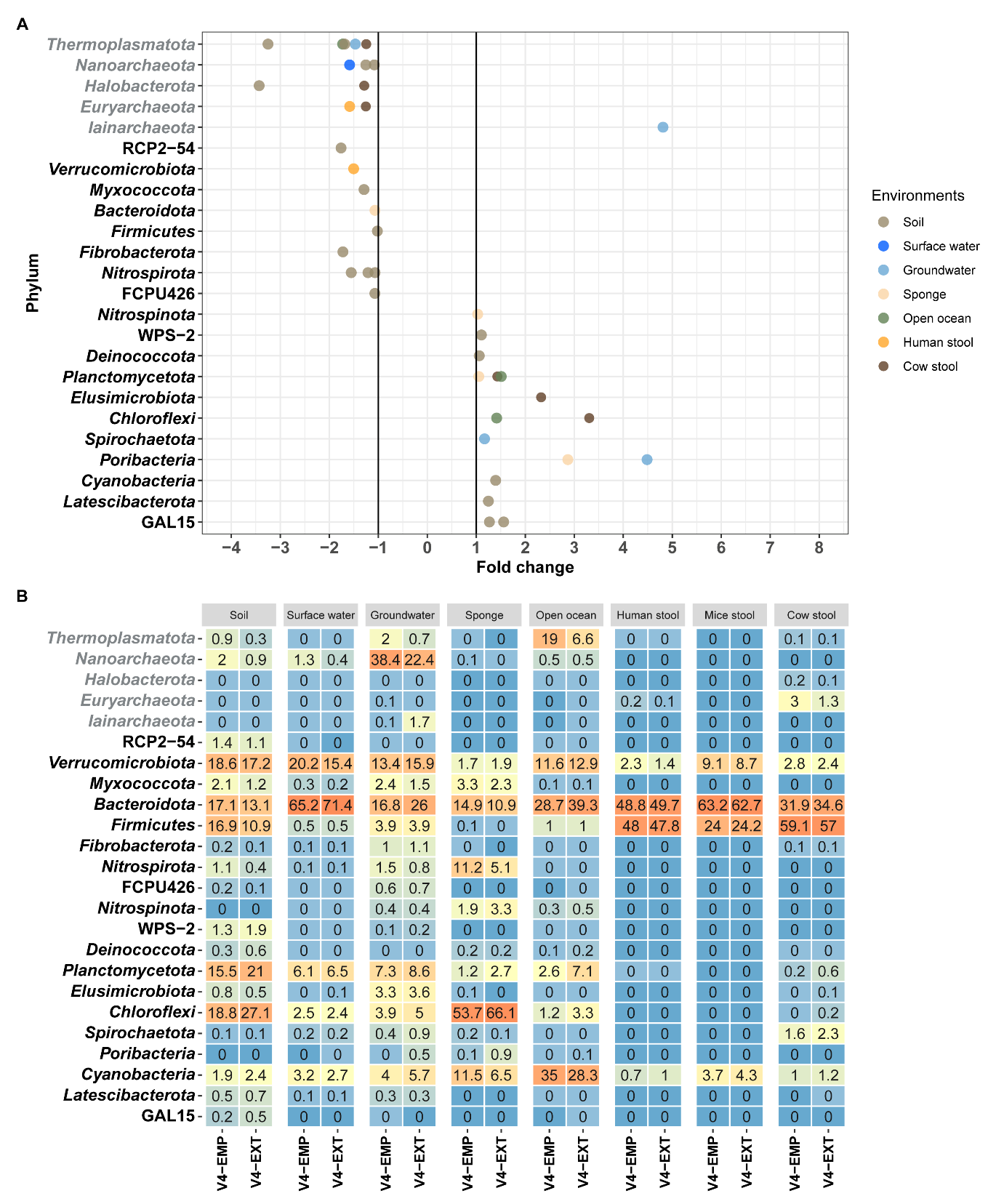


**Figure S2.** A) Fold change in the relative abundance of each phylum in datasets, grouped by source environment, generated using the V4-EXT versus the V4-EMP primes, after reads classified as *Patescibacteria* were removed. Phyla with a mean abundance >0.1% in any dataset are depicted. Phyla with a mean relative abundance of >0.1% for which mean relative abundance differences between datasets were above twofold are included. Fold change >1 indicates the phylum has a higher mean relative abundance in the V4-EXT dataset, and fold change <-1 indicates the phylum has a higher mean relative abundance in the V4-EMP dataset. B) Phyla level average relative abundance heatmap across different types of samples grouped by source environment, generated using the V4-EXT versus the V4-EMP primers, after reads classified as *Patescibacteria* were removed. Phyla with a mean relative abundance of >0.1% in either dataset are presented. Archaeal phyla are shown in grey font, and bacterial phyla are shown in black font.


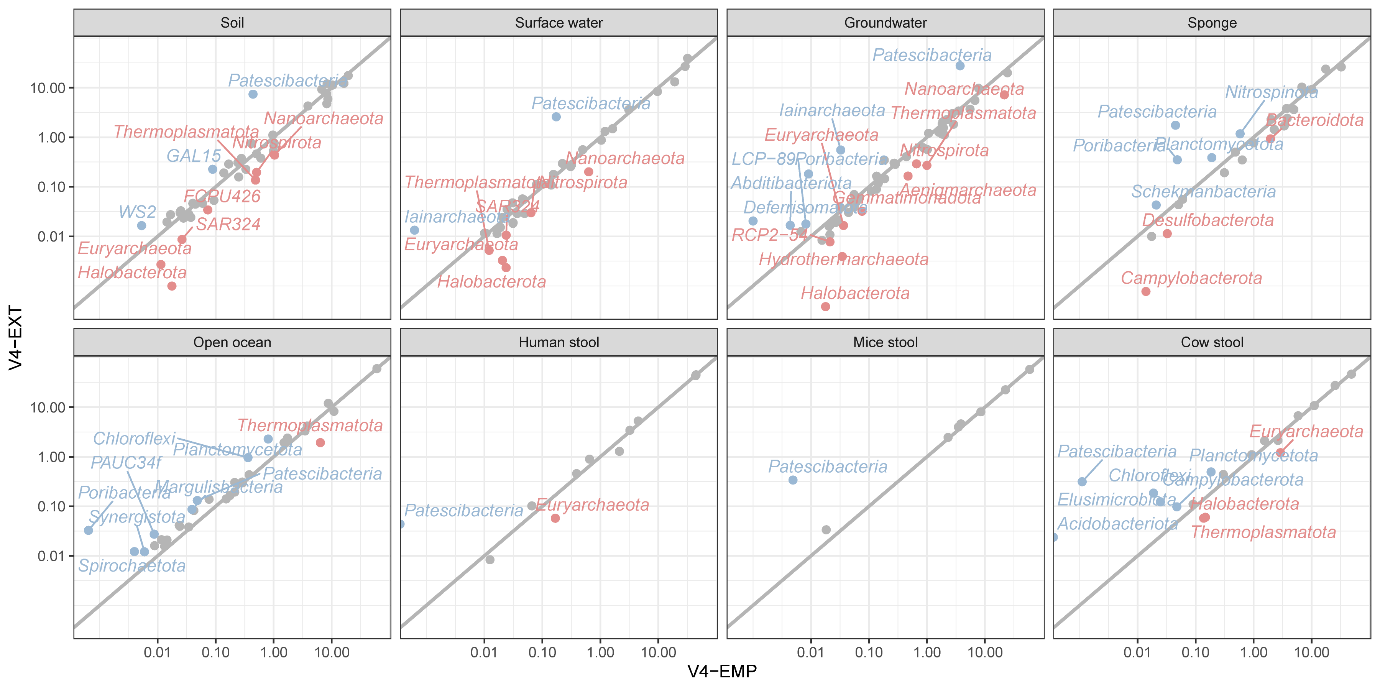


**Figure S3.** Comparison of the mean relative abundance (%) of phyla across different sample types in V4-EXT and V4-EMP datasets. Phyla with less than 0.01% average relative abundance in the V4-EMP or V4-EXT datasets are not depicted. Grey: phyla with less than twofold difference in relative abundance between datasets. Blue: phyla overrepresented by at least twofold with V4-EXT. Red: phyla overrepresented by at least twofold with V4-EMP. The phylum name SAR324 clade (Marine group B) is indicated as SAR324.


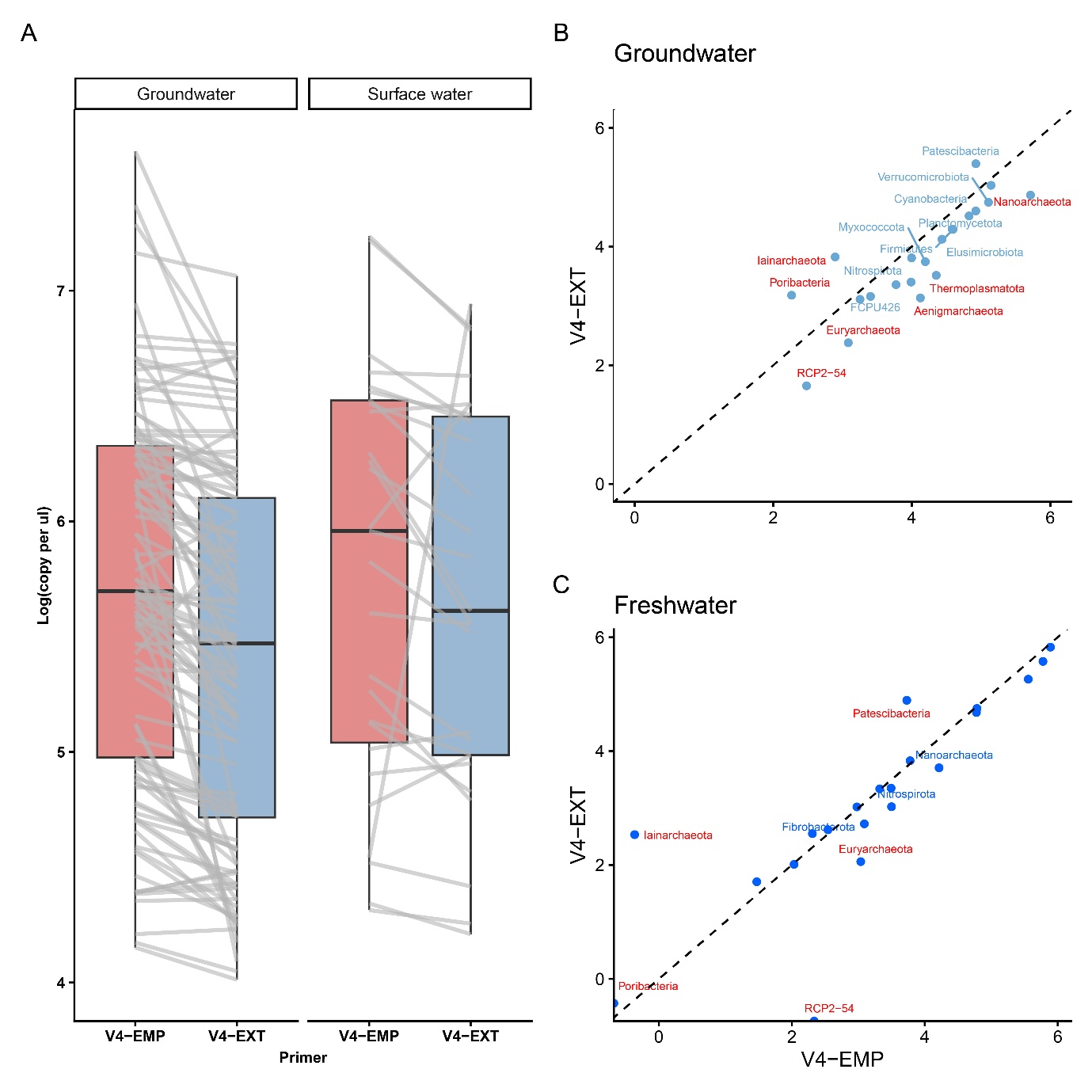


**Figure S4**. Absolute 16S rRNA copy number comparison at phylum level between two primer pairs. Compositional amplicon sequencing data was therefore normalized by dPCR-based 16S rRNA gene copy numbers. Panel A: copy number per microliter DNA extract as detected by dPCR (mitochondrial and chloroplast fraction has been removed before analysis); panel B and C: log5 fold mean absolute abundance comparison of phyla 16S rRNA gene copy numbers between the V4-EMP datasets (x-axis) and V4-EXT datasets (y-axis). Phylum with fold change value >1 or < -1 are shown with lightblue (Groundwater; panel B) and darkblue (Freshwater; panel C) labels; Phylum with log5 fold change value >1 or <-1 are shown with red labels.


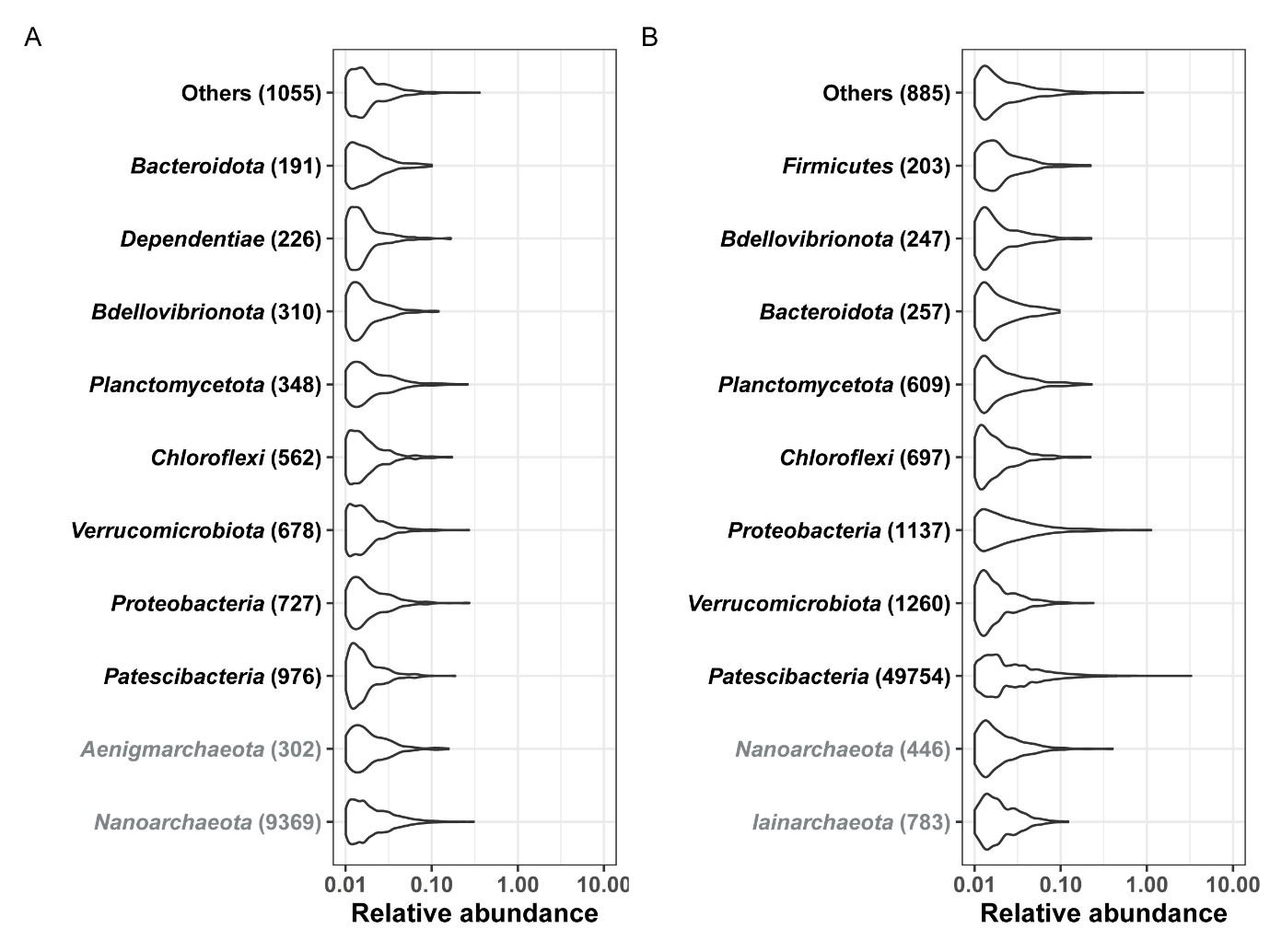


**Figure S5.** Phylum-level classification of ASVs (relative abundance>0.01%) without genus-level hits in the SILVA v138.2 database, generated by A) the V4-EMP B) V4-EXT primer pair. Each dot represents an observation of an ASV in a sample, and only observations with >0.01% relative abundance are shown. Per sample relative abundance is displayed on the x-axis, on a logarithmic scale. Top10 phyla are shown in the plot, while other phyla are collapsed in others. Archaeal phyla are shown in grey font, and bacteria phyla are shown in black font.


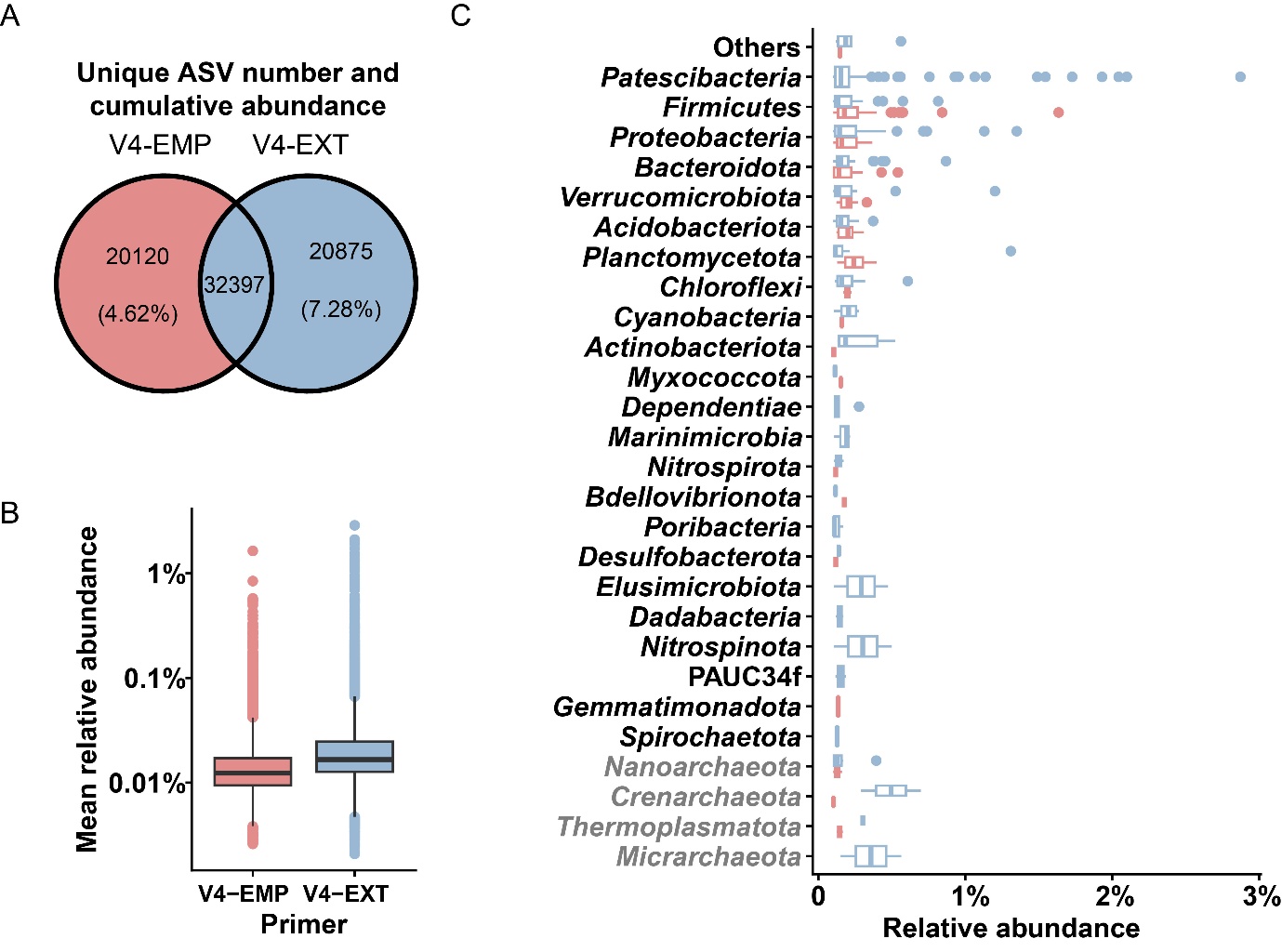


**Figure S6**. ASVs only detected by the V4-EMP (red) or V4-EXT (blue) primer pair. Panel A: Box plot of mean relative abundance of ASVs detected across samples uniquely by V4-EMP or V4-EXT primer pair. Panel B: Number of unique ASVs and their respective cumulative abundance detected by the V4-EMP or V4-EXT primer pair. Panel c: Phylum level classification boxplot of unique ASVs detected by the V4-EMP or V4-EXT primer pair with mean relative abundance > 0.1.

**Supplementary table legends**

**Table S1.** Summary of Sample JMF IDs and Dataset JMFS IDs, as well as their source habitats, used in this study.

**Table S2.** Taxonomy classification by the SILVA database and Greengene2 database.

**TableS3.** Percentage of ASVs in different habitats can not be mapped to the SILVA and Greengene2 database with >94.5% or >99% hits.

**TableS4.** Percentage of archaeal ASVs in different habitats can not be mapped to the SILVA and Greengene2 database with >94.5% or >99% hits.

**Table S5.** Phyla-specific in silico coverage of the three analyzed 16S rRNA gene V4 region primer pair versions using the SILVA database (SILVA_138.2_SSURef_NR99) as reference. Numbers indicate the percentage of sequences from each phylum in the SILVA database covered without mismatch or with one mismatch by the V4-EMP (Parada, Needham, and Fuhrman, 2016; Apprill et al., 2015), V4-CPR (Hu et al., 2024), and V4-EXT (this study) primers. Archaeal phyla are shown in grey font, and bacterial phyla are shown in black font.

**Table S6.** The percentage of sequences classified as chloroplast, mitochondrial, or eukaryotic 16S or 18S rRNA genes in the V4-EMP and V4-EXT datasets obtained from different sample types. The grey background and bold font indicates that a significant difference (pairwise Wilcoxon test, p<0.05) was detected between the V4-EMP and V4-EXT datasets.

**Table S7.** Fold changes in the relative abundances of each phylum in different datasets generated by the V4-EMP and V4-EXT primer pair originally and after Patescibacterial sequenced were removed.
